# Supplementary material for: Discovery and pharmacophoric characterization of chemokine network inhibitors using phage-display, saturation mutagenesis and computational modelling
Source: Nat Commun. 2023 Sep 16;14:5763. doi: 10.1038/s41467-023-41488-z (PMC10505172; doi:10.1038/s41467-023-41488-z)
Supplement: Supplementary file 4 — Source Data [file 41467_2023_41488_MOESM4_ESM.zip › SourceData/scripts/xNNKs_20230808183658.tar!/xNNKs/analysis/heatmaps/SV_HD2X_alanine_heatmap.pdf]

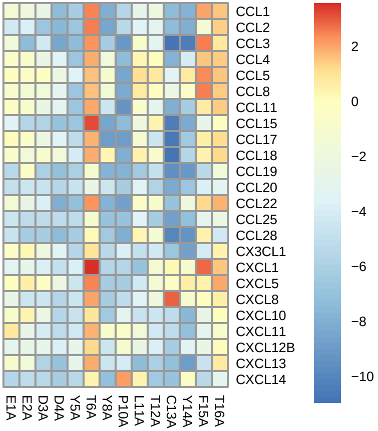

CCL1
CCL2
CCL3
CCL4
CCL5
CCL8
CCL11
CCL15
CCL17
CCL18
CCL19
CCL20
CCL22
CCL25
CCL28
CX3CL1
CXCL1
CXCL5
CXCL8
CXCL10
CXCL11
CXCL12B
CXCL13
CXCL14
E1A
E2A
D3A
D4A
Y5A
T6A
Y8A
P10A
L11A
T12A
C13A
Y14A
F15A
T16A
2
0
−2
−4
−6
−8
−10
